# Paving the way for more precise diagnosis of EcPV2-associated equine penile lesions

**DOI:** 10.1186/s12917-019-2097-0

**Published:** 2019-10-22

**Authors:** Anna Sophie Ramsauer, Garrett Louis Wachoski-Dark, Cornel Fraefel, Kurt Tobler, Sabine Brandt, Cameron Greig Knight, Claude Favrot, Paula Grest

**Affiliations:** 1 Institute of Virology, Vetsuisse Faculty, University of Zurich, Zurich, Switzerland; 2 Dermatology Department, Vetsuisse Faculty, University of Zurich, Zurich, Switzerland; 3 Department of Veterinary Clinical and Diagnostic Sciences, Faculty of Veterinary Medicine, University of Calgary, Calgary, Canada; 4 Research Group Oncology, Equine Clinic, University of Veterinary Medicine Vienna, Vienna, Austria; 5 Institute of Veterinary Pathology, Vetsuisse Faculty, University of Zurich, Zurich, Switzerland

**Keywords:** Equine papillomavirus type 2 (EcPV2), Squamous cell carcinoma (SCC), Papilloma, Benign hyperplasia, Carcinoma in situ (CIS), RNA-scope, Immunohistochemistry

## Abstract

**Background:**

There is growing evidence that equine papillomavirus type 2 (EcPV2) infection is causally associated with the development of equine genital squamous cell carcinomas (SCCs). Early stages of disease present clinically as plaques or wart-like lesions which can gradually progress to tumoural lesions. Histologically these lesions are inconsistently described as benign hyperplasia, papilloma, penile intraepithelial neoplasia (PIN), carcinoma in situ (CIS) or SCC. Guidelines for histological classification of early SCC precursor lesions are not precisely defined, leading to potential misdiagnosis. The aim of this study was to identify histologic criteria and diagnostic markers allowing for a more accurate diagnosis of EcPV2-associated equine penile lesions.

**Results:**

A total of 61 archived equine penile lesions were histologically re-assessed and classified as benign hyperplasia, papilloma, CIS or SCC. From these, 19 representative lesions and adjacent normal skin were comparatively analysed for the presence of EcPV2 DNA and transcripts using PCR and RNA in situ hybridisation (RISH). All lesional samples were positive by EcPV2 PCR and RISH, while adjacent normal skin was negative. RISH analysis yielded signal distribution patterns that allowed distinction of early (hyperplasia, papilloma) from late stage lesions (CIS, SCC). Subsequently, the 19 lesions were further assessed for expression of p53, Ki67, MCM7 and MMP1 by immunohistochemistry (IHC). All four proteins were expressed in both normal and lesional tissue. However, p53 expression was up-regulated in basal keratinocyte layers of papillomas, CIS and SCCs, as well as in upper keratinocyte layers of CIS and SCCs. MCM7 expression was only up-regulated in upper proliferating keratinocyte layers of papillomas, CIS and SCCs.

**Conclusion:**

This study proposes combining a refined histological protocol for analysis of equine penile lesions with PCR- and/or RISH based EcPV2-screening and p53/MCM7 IHC to more accurately determine the type of lesion. This may help to guide the choice of optimum treatment strategy, especially at early stages of disease.

## Background

Papillomaviruses (PVs) are a family of small viruses consisting of an icosahedral capsid harbouring a relatively short (≈8 kbp) and circular dsDNA genome. The latter contains ORFs for regulatory and capsid proteins, and importantly, for two to three oncoproteins, i.e. E6, E7, and frequently E5. PVs are usually host-specific and have a pronounced tropism for cutaneous and mucosal keratinocytes [1]. From the more than 200 human PV (HPV) types known today, at least 15 types have been recognized as carcinogenic viruses and thus are designated as high-risk HPVs (hrHPVs). Infection of humans with these types is responsible for nearly 100% of cervical cancers, about 50% of anogenital squamous cell carcinomas (SCCs) and 25% of head and neck SCCs [2].

Equine papillomavirus type 2 (EcPV2) was first identified from cases of equine genital carcinoma in situ (CIS) and penile SCCs in 2008, and its genome was fully characterized [3]. Since then, ample evidence has accumulated that EcPV2 infection is aetiologically associated with the development of equine genital SCCs [4].

SCCs arise from cutaneous and mucosal keratinocytes. In their initial stages, lesions may present either as whitish plaques or as exophytic wart-like lesions/papillomas with a cauliflower-like appearance. These may gradually progress to cancerous lesions like locally aggressive CIS and finally to invasive SCCs [5, 6]. The EcPV2 genome has been shown to reside in up to 100% of early and late lesions in an episomal and/or integrated form [4, 7, 8]. In a subset of lesions, infection also appears to be productive, as evidenced by immunocapture PCR-mediated detection of virion-like structures [7]. Importantly, EcPV2 transcribes its two oncogenes E6 and E7 at all stages of disease, which strengthens the evidence for an active involvement of this virus in progression of benign lesions to malignant neoplasms [4, 7, 8]. Given the presumed papillomaviral aetiology of equine genital lesions and their high potential for malignant transformation and metastasis, selection of the most appropriate therapeutic strategy and its outcome largely depends on early and accurate tumour diagnosis. In conformity with a grading system employed in human medicine [9], a system for the grading of equine penile SCCs has already been proposed in 2011 [10]. However, implementation of this system is partly hampered by the fact that the histological features of equine genital SCC precursor lesions are not clearly defined currently, potentially leading to misdiagnosis. In human oncology, difficulty in distinguishing hrHPV-induced benign lesions from more progressive lesions [11] has led to the establishment of more precise diagnostic criteria that rely not only on histological examination, but also on the screening of neoplastic tissue for HPV infection and expression of selected tumour markers [12, 13]. Such criteria have yet to be established for accurate diagnosis of EcPV2-associated equine tumours.

In human oncology, tumour suppressor protein p53 and cell proliferation-associated protein Ki67 are well established markers that allow for distinction between the different stages of cervical neoplasia [13]. Mini-chromosome maintenance proteins (MCMs) are over-expressed in high-grade human cervical lesions and are routinely used as surrogate markers of hrHPV oncoprotein activity [14]. Matrix metalloproteinases (MMPs) have a crucial role in both HPV-unrelated and hrHPV-driven tumour cell migration and invasion in that they orchestrate the degradation of the extracellular matrix and basement membranes [15, 16]. In the veterinary literature, overexpression of MMP1 in association with infiltrative growth has been reported for bovine papillomavirus type 1-induced equine sarcoids [17, 18], and canine oral melanoma [19]. On these grounds, the hypothesis of this study is that EcPV2 presence and transcription in concert with over-expression of some or all of these marker proteins could be indicative of progressive behaviour of EcPV2-associated equine penile lesions. To test this hypothesis, 61 archived equine penile lesions were first re-assessed on the basis of a refined histopathological protocol. Then 19 representative penile lesions were subjected to further analyses, i.e. screening for EcPV2 infection and transcription, and expression of p53, Ki67, MCM7 and MMP1.

## Results

### Histological criteria for lesion classification as benign hyperplasia, papilloma, CIS or SCC

**Fig. 1:**
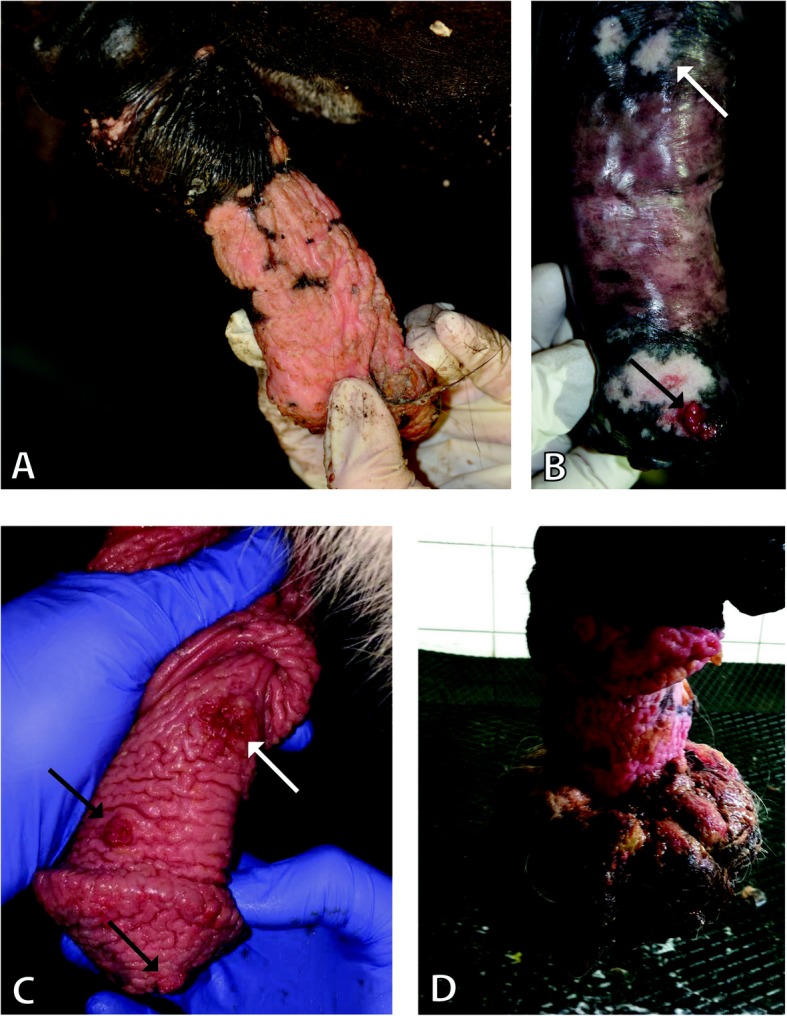
EcPV2 associated gross lesions. Representative images of EcPV2 associated gross lesions: **a**.) Depigmented plaques and hyperplastic epithelium of the penis shaft, developing up to the glans penis are visible. Histologically these lesions were diagnosed as benign hyperplasia (#19). **b** Two depigmented plaques can be seen on the penis shaft (white arrow) and one on the glans penis, where a small wart-like lesion (black arrow) is also visible, which was histologically diagnosed as papilloma. The depigmented plaques on the shaft (white arrow) were diagnosed as carcinoma in situ (#6). **c** The whole penis has depigmented thickened epithelium. Two wart-like lesions are also visible (black arrows), which were histologically diagnosed as papillomas (#13). On the penis shaft there is a small ulcerated area (white arrow), which is the second recurrence of a surgically removed SCC. Histologically this lesion was classified as early SCC (#5). Both lesions of this horse were included in this study, listed as separate cases. **d** The whole penis shows depigmented and thickened epithelium. There is a massive proliferation/mass on the glans penis. Histologically this tumoural lesion was diagnosed as SCC (#3)

Equine penile lesions (*n* = 61) were retrieved from tissue archives of the Vetsuisse Faculties of Zurich and Bern, Switzerland. According to available records, lesions were grossly diagnosed as generalized hyperplasia (Fig. 1a, c and d), depigmented plaques (Fig. 1a and b), wart-like (Fig. 1b and c), ulcerative (Fig. 1c), or tumoural lesions (Fig. 1d), based on their clinical appearance (Table 1). Histologically, the different lesions were diagnosed as benign hyperplasia, papilloma, CIS or SCC. Following histological re-assessment of the 61 tumour sections, 19 representative lesions (6 hyperplasias, 5 papillomas, 3 CIS and 5 SCCs) were subjected to down-stream analysis (Tables 1 and 2). The following criteria were used for histological classification and the representative lesions were classified as follows:

**Table 1:** Sample specification

Sample	Breed	Age	Clinical Description	Histological Diagnosis
1	Pony	18	**preputial tumoural lesion, 5 cm diameter**	SCC
2	German riding horse	20	**multiple depigmented wart-like lesions glans penis**	SCC
3	Haflinger	16	generalized hyperplasia of the penis with **tumoural changes on the glans penis, 10 cm diameter**	SCC
4	Welsh Pony	22	generalized hyperplasia of the penis with **tumoural changes on the glans penis, 5 cm diameter**	SCC early
5	Icelandic horse	17	generalized hyperplasia and **ulcerative lesions on the penis shaft**	SCC early
6	Icelandic horse	22	**multiple depigmented plaques glans penis** and penis shaft with wart-like lesion on glans penis	CIS
7	Bavarian Warmblood	16	**multiple depigmented plaques** and tumoural lesion penis	CIS
8	Icelandic horse	16	**multiple wart-like lesions penis shaft**	CIS
9	n.a.	13	**wart-like lesion penis shaft**	Papilloma
10	Icelandic horse	20	**tumoural lesion penis 2 cm diameter**	Papilloma
11	Lusitano	14	**multiple large wart-like lesions**	Papilloma
12	Shetland Pony	20	**multiple large wart-like lesions penis**	Papilloma
13	Icelandic horse	17	generalized hyperplasia and **wart-like lesions**	Papilloma
14	n.a.	8	**depigmented plaques penis**	Hyperplasia
15	Welsh Pony	13	**depigmented plaques penis**	Hyperplasia
16	Lusitano	15	**depigmented plaques penis**	Hyperplasia
17	Freiberger	13	**generalized hyperplasia** and ulcerative lesions **penis**	Hyperplasia
18	Irish Warmblood	14	**plaque-like lesion penis shaft**	Hyperplasia
19	Icelandic horse	22	**plaque-like lesions penis shaft and glans**	Hyperplasia

*n.a.* not available, clinical description: the parts of the lesions, which were further assessed histologically in this study, are marked in bold

**Table 2:** RISH and marker screening results in all samples

ID	Diagnosis	EcPV2 RISH		p53		MCM7		Ki67	
		GS	DNS	b	nb	b	nb	b	nb
1	SCC (G3)	pos	2	3	2	4	3	1	1
2	SCC (G2)	pos	4	4	3	4	2	0	0
3	SCC (G3)	pos	7	4	2	4	2	2	1
4	early SCC (G3)	pos	0	4	2	4	2	0	0
5	early SCC (G3)	pos	3	4	3	4	3	3	1
6	CIS	pos	1	4	3	4	3	3	2
7	CIS	(pos)	0	4	3	4	2	2	1
8	CIS	(pos)	0	2	2	4	3	2	2
9	Papilloma	pos	27	4	1	4	3	1	1
10	Papilloma	pos	0	4	1	4	2	3	1
11	Papilloma	pos	69	4	2	4	4	3	2
12	Papilloma	pos	8	4	1	4	2	2	1
13	Papilloma	pos	14	4	1	4	3	2	1
14	Hyperplasia	pos	98	2	1	4	1	1	1
15	Hyperplasia	pos	72	3	1	4	2	2	1
16	Hyperplasia	pos	27	2	1	4	2	2	1
17	Hyperplasia	pos	84	2	1	4	2	3	1
18	Hyperplasia	pos	7	4	1	4	2	2	1
19	Hyperplasia	pos	49	4	2	4	1	2	1
9a	normal adjacent skin	neg	0	2	1	4	1	1	0
12a	normal adjacent skin	neg	0	3	2	4	1	1	1
14a	normal adjacent skin	neg	0	2	1	4	2	2	1
15a	normal adjacent skin	neg	0	3	2	4	1	2	1
16a	normal adjacent skin	neg	0	2	1	4	1	2	1

Pos: positive; (pos): weak positive, neg: negative; b: basal layer; nb: non-basal layers; 0: negative; 1: < 10% of cells positive; 2: 10–50% of cells positive; 3: 51–90% of cells positive; 4: > 90% of cells positive

**Fig. 2:**
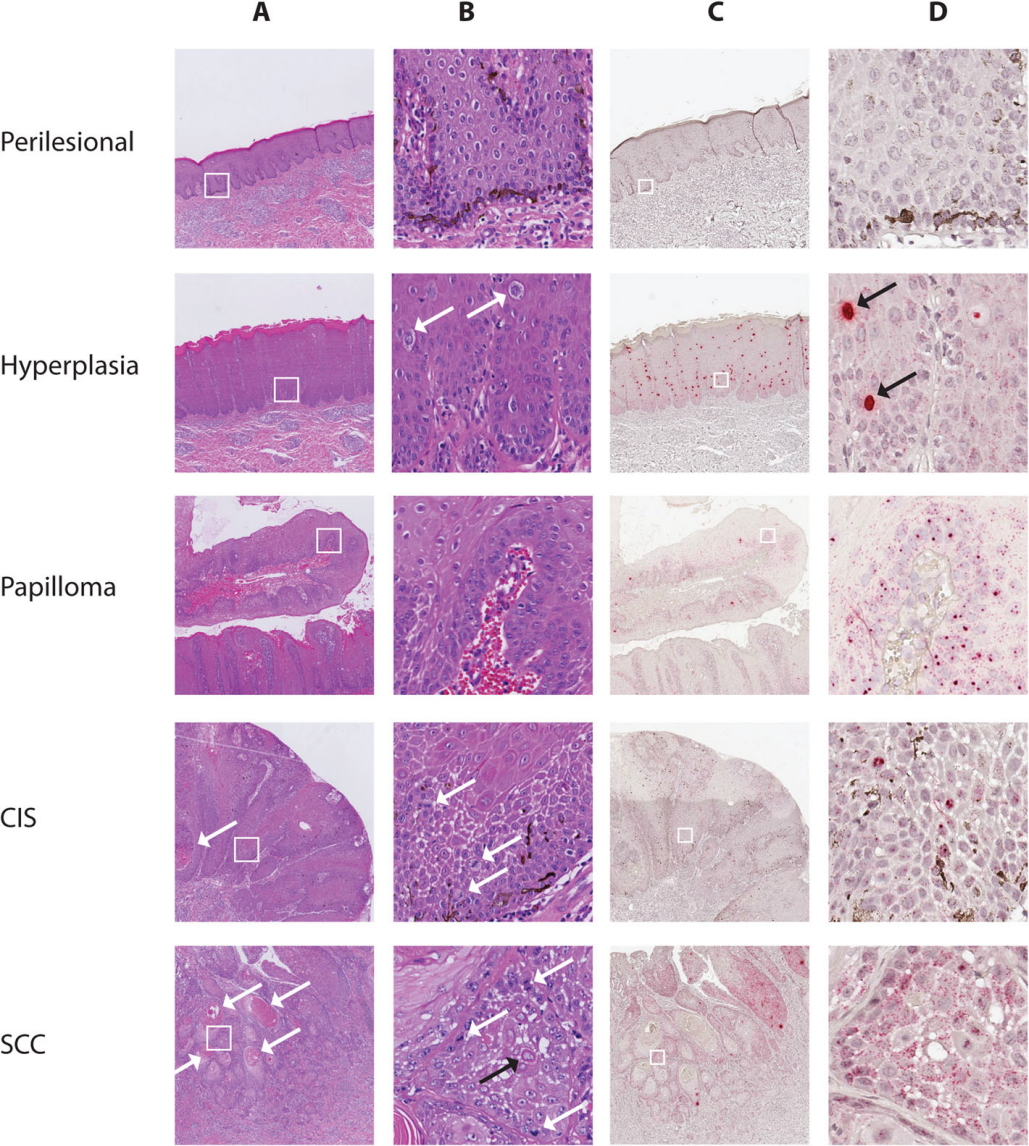
H&E staining and EcPV2 RISH signal distribution in penile lesions. One representative example per lesion (perilesional epithelium #14a, hyperplasia #14, papilloma #12, CIS #6 and SCC #5) is shown. **a** and **b**: H&E stained sections photographed using 2x (**a**) and 20x objectives (**b**). **c** and **d**: RISH stained sections photographed using 2x (**c**) and 40x (**d**) objectives. White squares in panel **a** and **c** mark the enlarged area shown in panel **b** and **d**. Perilesional skin: Squamous epithelium with normal maturation (**a** and **b**) and numerous pigmented cells in the basal layer (**b**). No viral signal is detectable in perilesional skin (**c** and **d**). Benign hyperplasia: Thickening of the epithelium and formation of broad rete ridges (**a**). There is orderly maturation and presence of koilocyte-like cells (white arrows) (**b**). Strong RISH signal is detectable, consisting of diffuse nuclear staining (DNS) (black arrows), which is detectable also at low magnification (**c** and **d**). The nuclear and cytoplasmic granular staining (GS) (small red dots in almost all lesional cells) is just visible at high magnification (**d**). Papilloma: Finger-like projection of moderately hyperplastic, mildly hyperkeratotic stratified squamous epithelium with normal differentiation and thin central cores of connective tissue (**a** and **b**). EcPV2 RISH staining in this case included mild DNS (**c**) and strong GS (**d**). CIS: Transepithelial mildly disrupted epithelial maturation can be seen (**a** and **b**). The keratinocytes show increased variability of keratinocytes and mitotic figures in the suprabasal region can be noted (white arrows **b**). There is intra-epithelial keratin pearl formation (white arrow **a**). EcPV2 RISH GS is just detectable at 40x magnification and no DNS is present (**c** and **d**). SCC: Infiltrating islands and trabeculae of moderately differentiated squamous epithelium with keratin pearl formation, (white arrows **a**). The keratinocytes are highly variable, show numerous mitotic figures (white arrows) and some cells are keratinized (black arrow) (**b**). The EcPV2 RISH DNS is rarely detectable within the infiltrating islands (**c**), while strong GS can be noticed (**d**)

Lesions were diagnosed as benign hyperplastic lesions when displaying thickening of the epithelium with orderly maturation and formation of broad rete ridges (Fig. 2a, b and Additional file 1: Figure S1). Six cases were included, all with a sharp transition from the normal to the hyperplastic epithelium. Hyperplastic lesions were evaluated for the presence of koilocyte-like cells typically presenting as swollen keratinocytes with a pale blue cytoplasm with or without intracytoplasmic dark blue granules (keratohyalin granules) and a normal or pyknotic nucleus (Fig. 2b). Two hyperplasias (case number (#) 15, #16) exhibited low numbers of koilocyte-like cells (a maximum of 5 at low magnification (10x objective)), two cases (#17, #19) contained moderate numbers (between 6 and 10) and another numerous (more than 10) koilocyte-like cells (#14). All six cases exhibited depigmentation, and focal to moderately extended areas with crowding of basal cells were observed. Keratin pearl formation within the hyperplastic epithelium was present in two cases (#15, #18).

Lesions were classified as papillomas when showing finger-like projections of moderately hyperplastic, usually mildly hyperkeratotic stratified squamous epithelium with normal differentiation and thin central cores of connective tissue (Fig. 2a, b and Additional file 1: Figure S1). Five cases all showing typical features were included. In one of these cases low to moderate numbers of koilocyte-like cells were present (#11).

Diagnosis of CIS was made in cases where transepithelial and abnormal epithelial maturation, increased variability of keratinocytes, and mitotic figures in the suprabasal region were noted (Fig. 2a, b and Additional file 1: Figure S1). None of the three included cases contained koilocyte-like cells. One had extensive basaloid differentiation of the keratinocytes (#8) and two had intraepithelial keratin pearl formation (#6, #8) (Fig. 2a). There were small areas with focal disruption of the basement membrane suspected to represent a microinvasion in two cases (#6, #7).

Lesions were classified as SCCs when they consisted of infiltrative islands and trabeculae of squamous epithelial cells with or without keratinization, originating from the epithelium and infiltrating the underlying dermal tissue (Figs. 2a, b and Additional file 1: Figure S1). In two cases, depth of infiltration was limited (less than 0.5 cm) and, therefore, these were classified as early infiltrative (#4, #5). All SCCs were mainly moderately differentiated, exhibiting keratin pearl formation, moderate nuclear and cellular pleomorphism, and obvious mitotic figures ranging from 10 to 30 per 10 high power fields. In four cases (#1, #3, #4, #5) histological examination revealed very poorly differentiated areas without keratinization located primarily at the tumour front. Therefore these cases were classified as grade 3, and one case (#2) as grade 2, corresponding to Van den Top 2015 [20]. Vascular invasion was detected in two cases (#2, #3). Hyperplasia of penile epithelium was always present adjacent to the SCC, and in two cases moderate to high numbers of koilocyte-like cells were observed within the hyperplastic areas.

### Depigmented plaques and wart-like lesions are not always benign

The mean age of the horses in this study is 17 years with 61% of the horses being ponies or small horses with a high proportion of Icelandic horses (28%) (Table 1). The histological diagnosis did not always correlate with the clinical appearance of the lesions. Benign hyperplasia was usually described clinically as depigmented plaques, plaque-like lesions or generalized hyperplasia of the penis. The clinical description for papillomas was usually wart-like lesions of different size, but was in one case also described as tumoural lesion. CIS cannot be distinguished from benign lesions from the clinical point of view. They can present as depigmented plaques (like benign hyperplasias) or wart-like lesions (like papillomas). SCCs are usually described as tumoural or ulcerative lesions, but a depigmented wart-like lesion may already be a SCC (Table 1). In conclusion, benign and malignant lesions cannot be reliably distinguished clinically but only by histological examination.

### EcPV2 DNA and oncogene transcripts were present in 100% of lesions tested

In the first step, DNA extracted from the 19 penile lesions (i.e. 6 benign hyperplasias, 5 papillomas, 3 CIS, and 5 SCCs) were assessed for the presence of EcPV2 DNA by specific EcPV2 E6 PCR. This yielded a positive result in all cases. To check for potential co-infection and the presence of other papillomaviruses associated with genital lesions in horses, broad-range PV PCRs were performed using CP4/5 and Fap59/64. No other PVs were detectable within these lesions. In the next step, lesions were tested for the presence and cellular localization of EcPV2 E6 and E7 transcripts using RISH. All 19 lesions (100%) were positive for viral E6 and E7 mRNA, while perilesional normal skin if present on the same slides, was negative (Table 2, Fig. 2c and d). RISH yielded two different signal distribution patterns: a finely scattered granular signal (GS) throughout the nucleus and cytoplasm, or, alternatively, an intense diffuse nuclear signal (DNS). Abundant GS was detectable in all penile lesions at high magnification (40x objective), while adjacent perilesional tissue did not contain signal (Table 2, Fig. 2c and d). In two CIS lesions, however, the GS was scarce. DNS, when present, was easily visible at low magnification (4x objective) and predominantly in the upper keratinocyte layers. A DNS was frequently detected in hyperplasias and papillomas, but rarely detectable in SCCs and CIS, and absent in perilesional tissue (Table 2, Fig. 2c, d and Additional file 2: Figure S2). As a consequence, the difference between the amount of DNS in benign (hyperplasia and papilloma) and malignant (CIS and SCC) lesions was significant (two-tailed Mann Whitney test *p* = 0.0011). Observed virally induced cytopathic changes, i.e. koilocyte formation, corresponded partially with DNS localization (Fig. 2, Additional file 1: Figure S1 and Additional file 2: Figure S2).

### Upper keratinocyte layers of CIS and SCCs exhibit up-regulated p53 and MCM7 expression

Following PCR and RISH screening, the 19 penile lesions were assessed for expression of the surrogate markers for proliferation and cancer p53, Ki67, MCM7, and MMP1 using IHC.

**Fig. 3:**
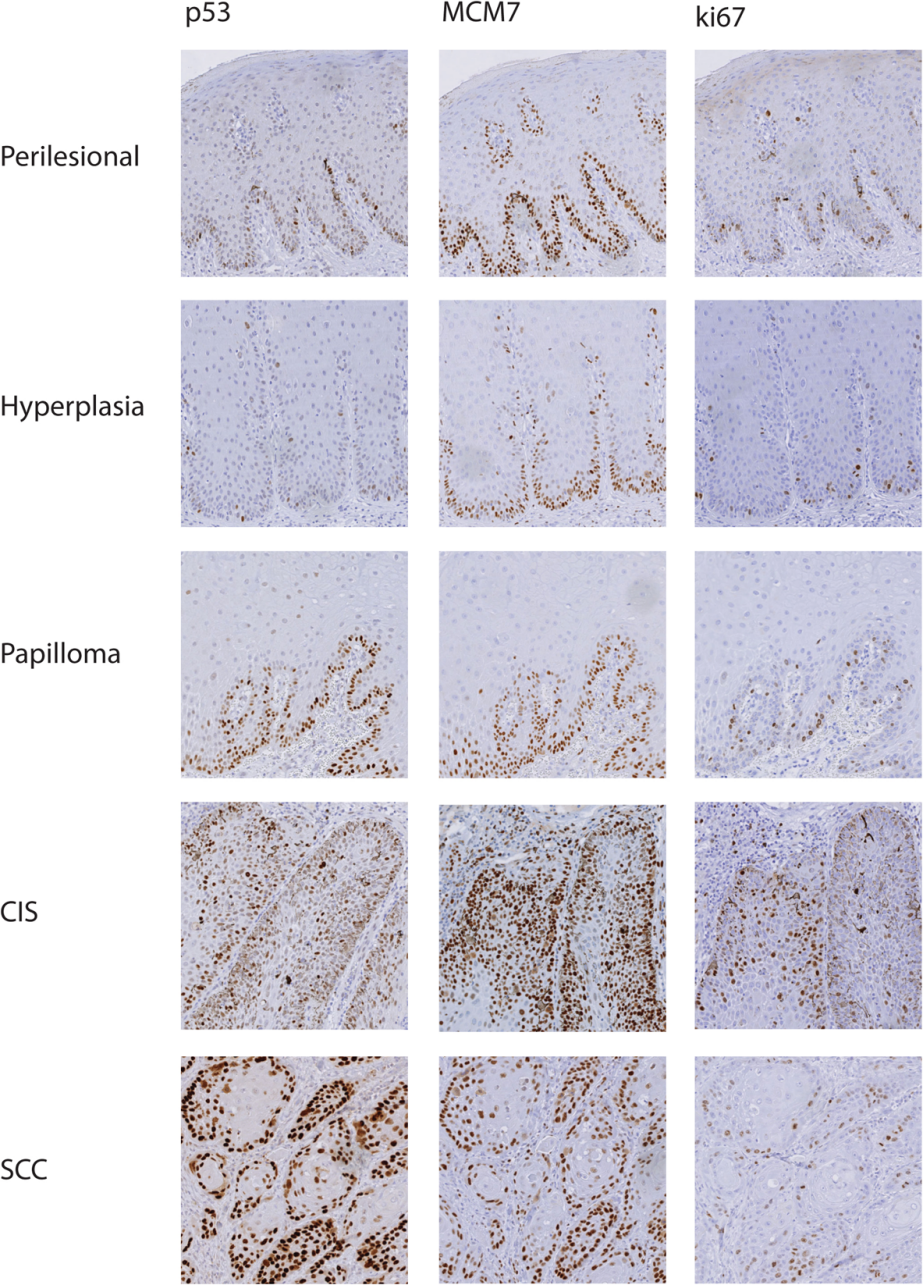
p53, MCM7 and Ki67 immunostaining of penile lesions. One representative example per type of lesion (perilesional normal epithelium #14a, hyperplasia #14, papilloma #12, CIS #6 and SCC #3) is represented. p53, MCM7 and Ki67 immunostaining was photographed using the 10x objective. p53 immunostaining in perilesional epithelium and hyperplasia is restricted mainly to the basal layer with nuclear labelling of few/single cells. In papilloma, CIS and SCC almost all basal cells express p53. However, in CIS and SCC p53 expression extended to the upper suprabasal layers and was enhanced. MCM7 is expressed in almost all basal cells within all lesional and perilesional tissue samples. In CIS and SCC, MCM7 is also expressed in upper suprabasal layers of the epithelium. Ki67 was restricted mainly to the basal layer with nuclear labelling of few/single cells of perilesional epithelium, hyperplasia and papilloma. In malignant lesions Ki67 expression was increased and extended to upper suprabasal layers

All lesions and adjacent normal tissue were positive for nuclear p53 immunostaining. In almost all SCCs, CIS and papillomas > 90% of basal cells expressed p53. In most hyperplasias and normal adjacent tissue, < 50% of basal cells were positive for this protein. In the majority of normal tissue and benign lesions, < 10% of non-basal keratinocytes were positive for p53. In CIS and SCCs, 10 to 90% of non-basal cells were positive for p53 (Table 2, Fig. 3 and Additional file 3: Figure S3).

In most of the lesions and normal perilesional tissue, > 10% of basal cells and <  10% of non-basal cells exhibited nuclear Ki67 expression. In two of three CIS however, Ki67 expression was more pronounced (> 10%). Unexpectedly, two SCCs appeared negative for this proliferation marker in the majority of the assessed fields (Table 2, Fig. 3 and Additional file 4: Figure S4).

Over 90% of basal cells in all tissues were positive for nuclear MCM7 immunostaining. In almost all normal perilesional tissues < 10%, and in most hyperplasias < 50% of non-basal cells expressed this protein. In SCCs, CIS and most papillomas, 10–90% of non-basal cells were positive for MCM7, depending on the degree of differentiation within the lesions (Table 2, Fig. 3 and Additional file 5: Figure S5).

MMP1-immunostaining of tissue sections yielded a diffuse cytoplasmic signal that was neither tumour- nor cell differentiation-specific and, therefore, was non-discriminatory (not shown).

## Discussion

### Proposal for histological classification of EcPV2-associated lesions

In horses SCC is reported to be the most common neoplasm of the external male genitalia [6, 21, 22]. It is suggested that the gross description plaques and wart-like lesions (papillomas) represent precursor lesions of this malignant tumour type [10, 21, 22]. However, this study shows, that plaques as well as wart-like lesions can already represent CIS i.e. malignant lesions preceding invasive SCCs and thus requiring more aggressive therapy. Therefore the histological analysis is very important in these lesions. However, gross and histological descriptions of these precursor lesions vary considerably in the literature [6, 10, 23–25]. When reassessing H&E-stained sections of equine penile lesions in this study, it was evident that the histological diagnosis varied among pathologists of the different institutions. This is likely due to the lack of a consistent and widely approved histological classification for EcPV2-associated equine penile lesions.

In humans, diagnosis of penile precancerous lesions and invasive carcinomas is likewise challenging [11]. Given that human penile precursor lesions and SCCs closely resemble their equine counterpart [7, 26], this study attempted to categorize EcPV2-associated equine penile lesions based on human data [9] and descriptions of equine disease [6, 10, 23–25] in order to obtain well defined histological classes regardless of their clinical appearance.

Thickening of the epithelium with orderly maturation accompanied by the formation of broad rete ridges corresponds to a (benign) hyperplasia as described by Chaux et al. [9]. In the equine literature the same features are sometimes attributed to papillomas in horses [6, 27]. Lesions with crowding of basal cells or small intraepithelial keratin pearls were included, because these alterations were considered to be dysplastic rather than neoplastic. Chaux et al. [9] use the term differentiated penile intraepithelial neoplasia (PIN) for such changes (with crowding of basal cells and/or intra-epithelial keratin pearls), whereby the specification “differentiated” implies a still benign, presumably non-neoplastic character of the change, which is why these two groups of lesions (hyperplasia and differentiated PIN) were combined in the present study.

The neoplastic alteration CIS can be distinguished by abnormal transepithelial maturation, increased variability of keratinocytes and frequent presence of mitotic figures in the suprabasal region. This description aligns with previous descriptions of equine penile CIS [24, 28] as well as with Chaux et al. [9] who refers to this change as a dedifferentiated PIN, indicating the increasingly malignant character. In the present study two CIS lesions had very small, solitary areas with a few cells focally disrupting the basement membrane, suspected to represent areas of microinvasion. This observation does not warrant a diagnosis of SCC, which should infiltrate profusely (over a large area, broadly) and not only in a very small area, but highlights the dynamic character and progressive potential of disease. To the best of our knowledge microinvasion is not mentioned in the equine literature on penile precursor and cancerous lesions. It is controversially discussed in regard to human cancerous lesions and rarely mentioned in the literature on penile carcinomas in man [9, 29, 30]. Chaux et al. [9] describes microinvasion in a differentiated PIN without then classifying the change as SCC.

Papillomas were diagnosed when finger-like projections of thickened, usually mildly hyperkeratotic squamous epithelium with normal maturation and thin central cores of connective tissue were present. This type of benign neoplastic lesion is not frequently mentioned in the literature and most closely corresponds to the papillomas described by Suarez-Bonnet et al. [26] and the fibropapillomas described by Lange et al. [24], whereas the papillomas described by Knight et al. [27] and van den Top et al. [6] more closely fit into our category of benign hyperplasia.

Lesions classified as SCCs contained infiltrative islands and trabeculae of squamous epithelial cells with or without keratinization originating from the epithelium and infiltrating profusely the underlying dermal tissue in accordance with van den Top, 2011 [10]. This study distinguishes between early invasive forms and late invasive forms, a distinction that is not normally found in the equine literature. Early invasive tumours (with limited infiltration of the sub-epithelial tissue) most likely represent an early stage of SCC. This information can be important for a clinician when deciding whether to choose a more aggressive or less aggressive therapeutic approach. SCCs were graded according to van den Top et al. [10] because prognosis is related to tumour grade. SCC can be heterogeneous and the poorest differentiated area determines the grade [20]. Most tumours of the present study were heterogeneous as described by others [10, 31] and consist of moderately as well as poorly differentiated areas and were therefore classified as grade 3. Interestingly, the only grade 2 SCC was described clinically as wart-like lesion, while the grade 3 SCCs were described clinically as tumoural lesions of larger sizes. However, since no follow-up information is available for the SCCs of this study and the number of cases is small, no further interpretation can be made.

### Aetiological association of EcPV2 with equine penile SCCs and precursor lesions based on PCR and RISH

As anticipated on the basis of previous reports reviewed by Sykora et al. [4] all lesions tested positive for EcPV2 DNA as revealed by virus type-specific PCR. Since there are other papillomaviruses described to be associated with equine genital lesions, and co-infections with other PVs were reported [32–34], also broad-range PV PCR using CP4/5 [35] and Fap 59/64 [36] primer sets was done to detect other potentially involved PVs. These primers are designed to also detect the equine PVs type 3–8 [32–34, 37] and the sarcoid associated bovine PVs type 1 and 2 [38]. Using these two broad-range PV primer sets, no other PVs were detectable, hence it is not likely that other PVs were involved in the development of the lesions assessed in this study. However, due to the lower sensitivity of broad-range primers compared to specific primers and the potential involvement of papillomaviruses which are not detectable by this primer sets, it cannot be excluded that other PVs play a role in some of these lesions. Nevertheless, it appears that EcPV2 is one of the main factors involved in the development of equine genital lesions, as it was detectable in all equine genital lesions assessed in this study.

Lesions were assessed also by EcPV2 E6/E7 RNA in situ hybridization (RISH) [39] to detect and localize viral nucleic acids. Of note, EcPV2 RISH recognizes both E6/E7 DNA and mRNA, possibly explaining the two different types of signal (GS and DNS) yielded by the method. Interestingly, RISH signal patterns analogous to those observed here in the EcPV2-associated equine genital lesions have been detected with hrHPV-associated neoplasms as well [40, 41]. Previous studies support the concept that DNS can be attributed to the presence of both papillomaviral RNA and DNA [12, 41]. One study proposed that DNS may result from probe hybridization with ‘unzipped’ single-stranded HPV-DNA, which occurs during episome synthesis in the productive phase of the HPV life-cycle. In that study, the authors proposed that RISH could be used for grading HPV-associated cervical intraepithelial neoplasia (CIN1–3) lesions, as CIN1 lesions exhibit abundant DNS, while CIN3 lesions exhibit little to no DNS in the transformative phase [12]. Similarly, in the present equine study benign lesions had a high level of DNS, suggestive of a productive EcPV2 life cycle, while CIS and SCC had a low level of DNS, as expected for transformative processes.

Importantly, histological reassessment of some lesions, which have previously been macroscopically interpreted as (benign) plaques, led to a diagnosis of CIS. The abundance of DNS helped distinguishing these malignant lesions from benign hyperplasia and is thus proposed as an adjuvant diagnostic marker. These findings also make clear that plaques do not necessarily represent early benign lesions and thus require careful diagnosis and corresponding treatment.

In this study 100% of lesions exhibited GS in accordance with EcPV2 PCR results. The perilesional normal skin surrounding the assessed lesions, if present on the same slide, was negative for EcPV2 RISH. It is known that EcPV2-DNA can also be detected in healthy horses with a prevalence between 1 and 18% in different studies [22, 42, 43], and that subclinical infections also might get cleared by the immune system, as up to 15–36% of healthy horses have antibodies against EcPV2 as well [42, 44]. However, looking at the results obtained in this study, it seems that if the horses develop lesions, this occurs exclusively in EcPV2 infected and transcriptionally active areas but not in EcPV2 negative areas. This finding further supports the concept that EcPV2 infection has an active role in tumour onset and progression [4].

In summary, RISH can be used to detect and localize EcPV2 RNA/DNA in FFPE tissue samples. The different signal distribution patterns and amount of DNS might help to distinguish between early proliferative and late progressive tumour stages, as the amount of DNS significantly differed between benign and malignant lesions.

### IHC-based marker gene expression in different lesions

p53 has a critical role in cell cycle regulation and is involved in cell cycle arrest, DNA repair and apoptosis [45]. In approximately 50% of human tumours, the p53 gene is mutated. Mutations within the p53 gene frequently compromise the tumour-suppressive activity of the corresponding protein, thus promoting tumour onset and progression [13]. In contrast to wild-type p53, which is an unstable protein with a short half-life, mutant p53 can accumulate within tumour cells, thus representing a potential tumour marker [46]. p53 immunopositivity has also been shown to strongly correlate with p53 mutational status [47]. However, anti-p53 antibody usually recognizes both accumulated wild-type and mutated forms of the protein [46]. This fact explains why the use of p53 as human cervical cancer marker is controversial [13]. In human penile SCCs however, strong nuclear p53 immunostaining has been consistently detected, in contrast to adjacent normal perilesional skin, which has a moderate to weak signal mainly confined to the basal layer [48]. In addition, p53 immunostaining can be used to distinguish human penile intraepithelial lesions from squamous hyperplasia [49] and p53 expression levels in human penile carcinomas may have prognostic value in predicting lymph node metastasis and survival [50]. In an equine study on penile lesions it was already shown, that expression of p53 increases with decreased differentiation of the tumour, but could not be used as prognostic factor for metastatic disease [20]. In the present equine study, lesional and histologically normal perilesional tissue were positive for p53. Given that, to date, equine p53 has not been assessed for mutations in the context of penile tumour disease, it is unclear whether wild-type and/or mutant p53 are detected by IHC. Nonetheless, p53 expression by basal cells of most SCCs, CIS and papillomas (> 90%), markedly differed from that of most hyperplasia and normal perilesional tissue (< 50%). Within the non-basal layers, < 10% of cells were positive for p53 in most perilesional, hyperplastic and papilloma tissues, while distinctly more non-basal cells expressed p53 in CIS and SCCs (10–90%). Consequently, assessing the number and locations of p53-positive cells within equine penile lesions might help to distinguish between benign and malignant lesions.

Ki67 is widely used in routine tumour assessment as a prognostic and predictive indicator [51]. In human cervical lesions, Ki67-staining is used for the grading of progressive cervical tumours [13]. In human penile cancer, Ki67 expression is correlated with tumour grade, although it has no prognostic value [52]. Based on this, the hypothesis of this study was that Ki67 may represent a potential diagnostic marker in horses. However, Ki67 staining of equine penile lesions yielded inconsistent results, with two SCCs appearing almost negative for this proliferation marker. Standardized tissue sample handling may be an important prerequisite for accurate assessment of the Ki67 index. Using the same Ki67 antibody, it was shown that insufficient, delayed and prolonged fixation can have a negative effect on Ki67 antibody binding [53]. The two negative/weak SCC samples were received from Germany and had both a prolonged fixation time, which might explain these results. When collecting horse samples from different regions, these procedures cannot always be standardized. Consequently, Ki67-staining cannot be recommended for diagnosis and lesion assignment of EcPV2-associated equine penile lesions. This is also supported by another study using this antibody in equine penile and preputial SCCs whereby they could also not find expression differences between different subtypes [20]. The proliferation marker MCM7 is more sensitive in detecting cycling cells than Ki67 in humans and dogs and therefor might be superior to Ki67 as a proliferation marker [54–56].

High expression of MCM7 can serve as a predictive biomarker for poor prognosis in human cancers [57]. In hrHPV-induced high-grade lesions, MCMs are over-expressed and serve as established surrogate markers for papillomaviral E6/E7 oncoprotein activity [14]. In particular MCM7 constitutes a reliable human cervical cancer marker. In normal and benign hyperplastic cervical tissue, MCM7 expression is restricted to basal and immediate para-basal layers, while full thickness immunostaining is observed in high grade cervical dysplasia and invasive cancers [58]. In the present study, lesional as well as normal perilesional basal keratinocytes expressed MCM7, as expected on the basis of human data. In non-basal keratinocyte layers, MCM7 expression was most often increased in papillomas, CIS and SCCs, depending on the degree of differentiation within the lesions. In non-differentiated parts of the lesions, cells appeared to be still proliferating and express MCM7 in higher levels, than in more differentiated parts of the lesion. In human cervical cancer, MCM expression is thought to depend on hrHPV E7 expression, which results in the inactivation of pRB and the related proteins p107 and p130. This in turn activates the E2F family of transcription factors and thereby induces expression of E2F-responsive genes such as MCMs [58]. Interestingly, the EcPV2 E7 open reading frame lacks a pRB binding domain [3, 4]. Hence, the mechanisms by which EcPV2 infection promotes MCM7 expression by non-basal cells remains unclear. Importantly, it has been shown that HPV16 can likewise induce E2F responsive genes - especially MCM7 - in the absence of E7 in reproductive epithelia and tumours [59]. In analogy, it can be speculated that MCM7 expression in upper keratinocyte layers of progressive penile lesions may be driven by EcPV2 E6. The RISH data generated in the present equine study do not fully support this possibility, as MCM7 expression and signals obtained by E6/E7 RISH did not always colocalize. In humans, MCM7 expression is also seen in HPV-negative penile cancers, suggesting that MCM7 expression may depend on the amount of proliferating cells within the lesions and not entirely on the papillomaviral oncoproteins E6 and E7 [60]. Gene expression profiling of EcPV2-associated equine genital cancer has shown a strong up-regulation of MCM transcripts [8]. In the present study, this finding has been confirmed on the protein level. Evaluation of MCM7 expression in upper epithelial layers may therefore help to distinguish well-differentiated equine penile lesions from less-differentiated, more proliferative lesions.

MMP1 transcription was recently shown to be upregulated in EcPV2-associated genital SCCs [8], and over-expression of this proteinase has been previously demonstrated to correlate with progression of bovine PV-induced equine sarcoids [17, 18]. However, in the present study MMP1-immunostaining of equine penile lesions yielded a diffuse cytoplasmic signal throughout the different cell layers of all samples, disqualifying MMP1 IHC or the staining protocol applied in the present study as a useful technique for investigation of EcPV2-associated penile lesions.

## Conclusions

Histological diagnoses of equine penile SCC precursor lesions are challenging since no consistent or validated classification system exists. In this study, histological criteria that allow for a more precise, and hence more consistent diagnosis of penile hyperplasias, papillomas, CIS and SCC, were developed and proposed. Importantly, it was also demonstrated that the general term ‘plaque’ is not synonymous with benign hyperplasia, as it may also describe CIS. Assessment of lesions for genomic EcPV2 sequences and transcripts proved useful for the discrimination of SCCs and associated precursor lesions from other penile tumour types such as sarcoids and melanomas and may help to distinguish between early proliferative and late progressive tumour stages. In addition, p53- and MCM7-immunostaining may provide additional information on the severity of a lesion as it may facilitate the distinction of benign lesions from more progressive EcPV2-associated penile tumours.

## Methods

### Sample material

The databases of the Institute of Veterinary Pathology of the Vetsuisse Faculty University of Zurich and the Institute of Animal Pathology of the Vetsuisse Faculty University of Bern were searched for penile SCC precursor lesions and SCCs. Sixty-one H&E-stained tumour sections were retrieved and histologically reassessed by microscopy. Following histological analysis, 19 representative snap-frozen and/or paraffin-embedded tumour samples diagnosed as benign hyperplastic lesions (*n* = 6), papillomas (*n* = 5), CIS (*n* = 3) and SCCs (*n* = 5) were chosen for further assessment in this study. Age, breed, clinical description from the referring clinicians and histological diagnosis of the included horses are shown in Table 1. It should be noted that in some horses more lesions were present simultaneously, while the lesion assessed in this study is marked in bold in Table 1. From one horse with an ulcerative lesion on the penis shaft and papillomas on shaft and glans penis, both lesions were included in this study and listed as separate cases (#5, #13). The images from perilesional normal skin were taken from five horses with papilloma or hyperplasia (# 9, #12, #14, #15, #16), which are also included in the study. In these cases also normal skin was present on the same slide. Therefore, the samples from normal adjacent skin were numbered as #9a, #12a, #14a, #15a and #16a (Table 1).

### PCR-based assessment of lesions for the presence of PV DNA

Total DNA was extracted from ±2 mm^3^ native, or 3 × 30 μm paraffin-embedded tumour specimens using a QIAamp DNA Minikit or QIAamp DNA FFPE Tissue Kit following the manufacturer’s instructions (Qiagen, Hilden, Germany). PCR-compatibility of extracted DNA was successfully confirmed by equine GAPDH PCR as described previously [33]. Subsequently, DNA aliquots were subjected to EcPV2-specific PCR using an EcPV2 specific primer set (E6-41f /E6–435) for amplification of the E6 gene (position 41–435) [3]. To assess whether also other PVs are present in those lesions, two other broad-range PV PCR reactions using the primer sets CP4/5 and Fap59/64 were [35, 36] analysed. Reactions were performed in 25 μl-volumes, containing 12 μl of REDTaq ReadyMIX (Sigma-Aldrich; Merck KGaA, Darmstadt, Germany), 8 μl of water, 2 μl of each forward and reverse primer (10 μM each) and 1 μl of extracted DNA as template. The cycling program consisted of a denaturation step of 3 min at 94 °C, followed by 40 cycles of 30 s at 94 °C, 30 s at 55 °C (ecGAPDH and EcPV2) or 42 °C (CP4/5 and FAP59/64) and 30 s at 72 °C. Then, amplicons were analysed by 1% TAE-gel electrophoresis and visualized by GelRed® Nucleic Acid Gel staining (Biotium; VWR International AG, Dietikon, Switzerland).

### RISH-based assessment of lesions for the presence and localization of EcPV2 E6 and E7 transcripts

Presence and localization of EcPV2 transcripts were assessed by E6/E7 RNA in situ hybridisation (E6/E7 RISH) according to Zhu et al. [41]. RISH was run on all FFPE tissue samples in replicates (5 per lesion) using the RNAscope 2.5 HD Detection Kit RED according to the manufacturer’s instructions (Advanced Cell Diagnostics, Newark, NJ, USA) with minor modifications: Xylene Substitute (Sigma-Aldrich) was used instead of xylene; slides were placed into boiling 1x target retrieval solution for 15 min to expose nucleic acids; counterstaining was performed in 20% instead of 50% haematoxylin (Sigma-Aldrich).

According to observed hybridisation patterns, distinction was made between a finely scattered GS throughout the nucleus and cytoplasm, and an intense DNS filling the entire nucleus (Fig. 1b). Lesions were classified as RISH-positive when GS-positive keratinocytes were detected by screening of ten high magnification fields (40x objective). The presence and number of DNS-positive cells were evaluated by counting in five low magnification fields (4x objective). The mean number of DNS-positive cells per field was evaluated and used to compare early (benign hyperplasia, papilloma) with advanced stages of disease (CIS and SCC). To determine the statistical significance of observed differences, a two-tailed Mann Whitney test using GraphPad Prism 7 (GraphPad Software, San Diego, CA, USA) was carried out.

### Immunohistochemical analysis of tumour sections for p53, Ki67, MCM7 and MMP1 expression

Five 3.5-μm sections per lesion were prepared from paraffin-embedded tissue and placed on positively charged glass slides. Immunohistochemical (IHC) staining was performed using an automated staining device (Dako Autostainer; Dako Schweiz GmbH, Basel, Switzerland) with diaminobenzidine (DAB) or 3-Amino-9-ethylcarbazole (AEC) (both from Dako) serving as chromogen and Meyer’s haematoxylin as counterstain. Negative controls were generated by omitting the primary antibody. Detailed incubation conditions for each antibody (anti-p53, −MCM7, −Ki67 and -MMP1) and respective providers are given in Table 3. For scoring purposes, the number of immunopositive cells in the basal cell layer was counted (if crowded, the next 1–2 layers were included) and percentages calculated. Using the same technique, the number of immunostained cells in the non-basal layers was also evaluated. The following scores (0–4) were assigned to calculated percentages of immunopositive cells: 0% = 0, < 10% = 1, 10–50% = 2, 51–90% = 3, > 90% = 4. Ten fields per tumour section were evaluated using the 10x objective and the mean scores per section calculated as represented by Fig. 1. Normal perilesional regions within tissue sections served as additional internal negative controls.

**Table 3:** IHC antibody specifications

Primary antibody	Use	Retrieval	2nd antibody	Chromogen	Control
Monoclonal mouse anti-Ki67 (clone MIB-1; Dako)	1:50 1 h, RT	98 °C; pH 9	REAL kit (Dako, K5007)	AEC	Equine skin, rectum
Monoclonal mouse anti-p53 (clone DO1; Santa Cruz)	1:100 1 h, RT	98 °C; pH 9	Envision mouse (Dako, K4001)	DAB	Equine SCC
Monoclonal mouse anti-MCM7 (clone DCS-141.2; Santa Cruz)	1:400 1 h, RT	98 °C; pH 9	Envision mouse (Dako, K4001)	DAB	Equine lymph node
Polyclonal rabbit anti-MMP1 (ThermoFisher)	1:50 O/N, 4 °C	98 °C; pH 7	Envision rabbit (Dako, K4003)	DAB	Equine SCC

*RT* Room temperature, *O/N* overnight, *AEC* Aminoethylcarbazol, *DAB* Diaminobenzidine

## Supplementary information

**Additional file 1: Figure S1.** H&E staining in all samples. One representative H&E staining image photographed using 4x objectives of each sample used in this study are shown (#1–3 SCC, #4, #5 early SCC, #6–8 CIS, #9–13 Papilloma, #14–19 Hyperplasia, #9a, #12a, #14a, #15a and #16a normal adjacent skin).

**Additional file 2: Figure S2.** RISH signal distribution in all samples. One representative RISH stained image photographed using 4x objectives of each sample used in this study are shown (#1–3 SCC, #4, #5 early SCC, #6–8 CIS, #9–13 Papilloma, #14–19 Hyperplasia, #9a, #12a, #14a, #15a and #16a normal adjacent skin).

**Additional file 3: Figure S3.** p53 immunostaining in all samples. One representative p53 immunostaining image photographed using 4x objectives of each sample used in this study are shown (#1–3 SCC, #4, #5 early SCC, #6–8 CIS, #9–13 Papilloma, #14–19 Hyperplasia, #9a, #12a, #14a, #15a and #16a normal adjacent skin).

**Additional file 4: Figure S4.** Ki67 immunostaining in all samples. One representative Ki67 immunostaining image photographed using 4x objectives of each sample used in this study are shown (#1–3 SCC, #4, #5 early SCC, #6–8 CIS, #9–13 Papilloma, #14–19 Hyperplasia, #9a, #12a, #14a, #15a and #16a normal adjacent skin).

**Additional file 5: Figure S5.** MCM7 immunostaining in all samples. One representative MCM7 immunostaining image photographed using 4x objectives of each sample used in this study are shown (#1–3 SCC, #4, #5 early SCC, #6–8 CIS, #9–13 Papilloma, #14–19 Hyperplasia, #9a, #12a, #14a, #15a and #16a normal adjacent skin).

## Data Availability

All data generated or analysed during this study are included in this published article and its supplementary information files.

## Abbreviations

#: Case number
CIN: Cervical intraepithelial neoplasia
CIS: Carcinoma in situ
DNS: Diffuse nuclear signal (by RISH)
EcPV2: Equine papillomavirus type 2
GS: Granular signal (by RISH)
H&E: Haematoxylin and eosin
HPV: Human papillomavirus
hrHPV: High risk human papillomavirus
IHC: Immunohistochemistry
MCM: Mini-chromosome maintenance protein
MMP: Matrix metalloproteinase
PIN: Penile intraepithelial neoplasia
PV: Papillomavirus
RISH: RNA in situ hybridisation
SCC: Squamous cell carcinoma
