# Supplementary figures and images for: Paving the way for more precise diagnosis of EcPV2-associated equine penile lesions
Source: BMC Vet Res. 2019 Oct 22;15:356. doi: 10.1186/s12917-019-2097-0 (PMC6805557; doi:10.1186/s12917-019-2097-0)

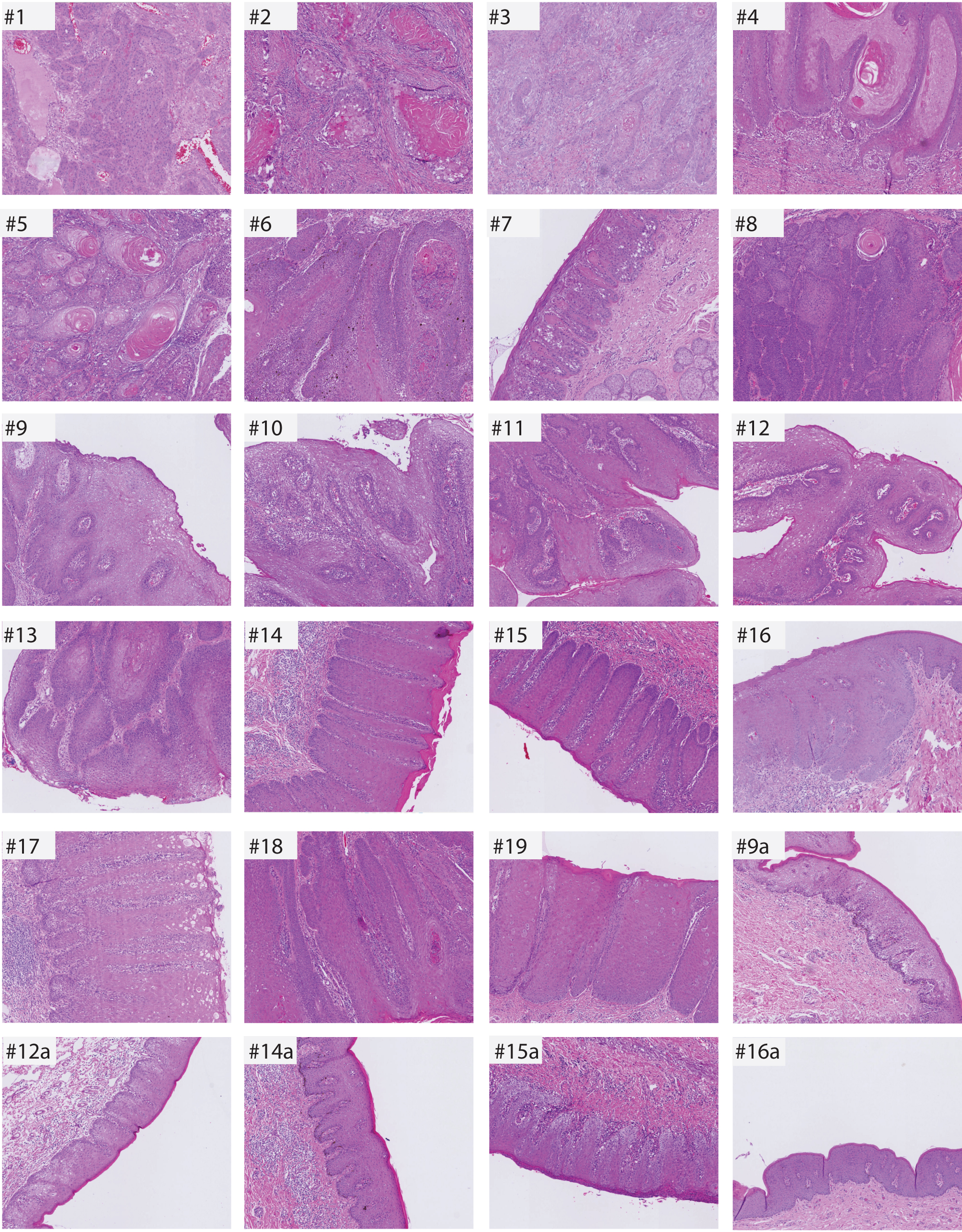

Supplement: Supplementary file 1 — Additional file 1: Figure S1. H&E staining in all samples. One representative H&E staining image photographed using 4x objectives of each sample used in this study are shown (#1–3 SCC, #4, #5 early SCC, #6–8 CIS, #9–13 Papilloma, #14–19 Hyperplasia, #9a, #12a, #14a, #15a and #16a normal adjacent skin). [file 12917_2019_2097_MOESM1_ESM.jpg]

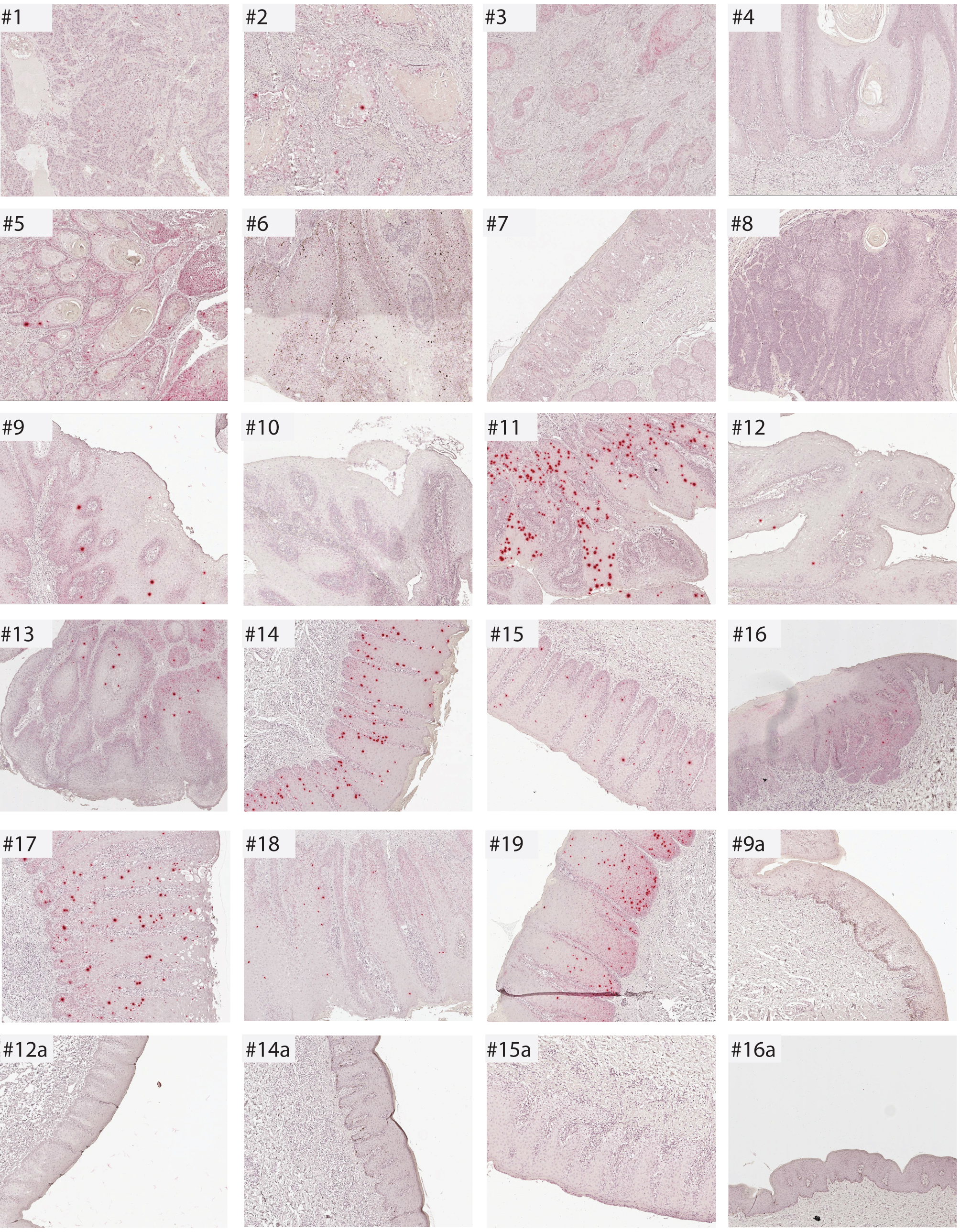

Supplement: Supplementary file 2 — Additional file 2: Figure S2. RISH signal distribution in all samples. One representative RISH stained image photographed using 4x objectives of each sample used in this study are shown (#1–3 SCC, #4, #5 early SCC, #6–8 CIS, #9–13 Papilloma, #14–19 Hyperplasia, #9a, #12a, #14a, #15a and #16a normal adjacent skin). [file 12917_2019_2097_MOESM2_ESM.jpg]

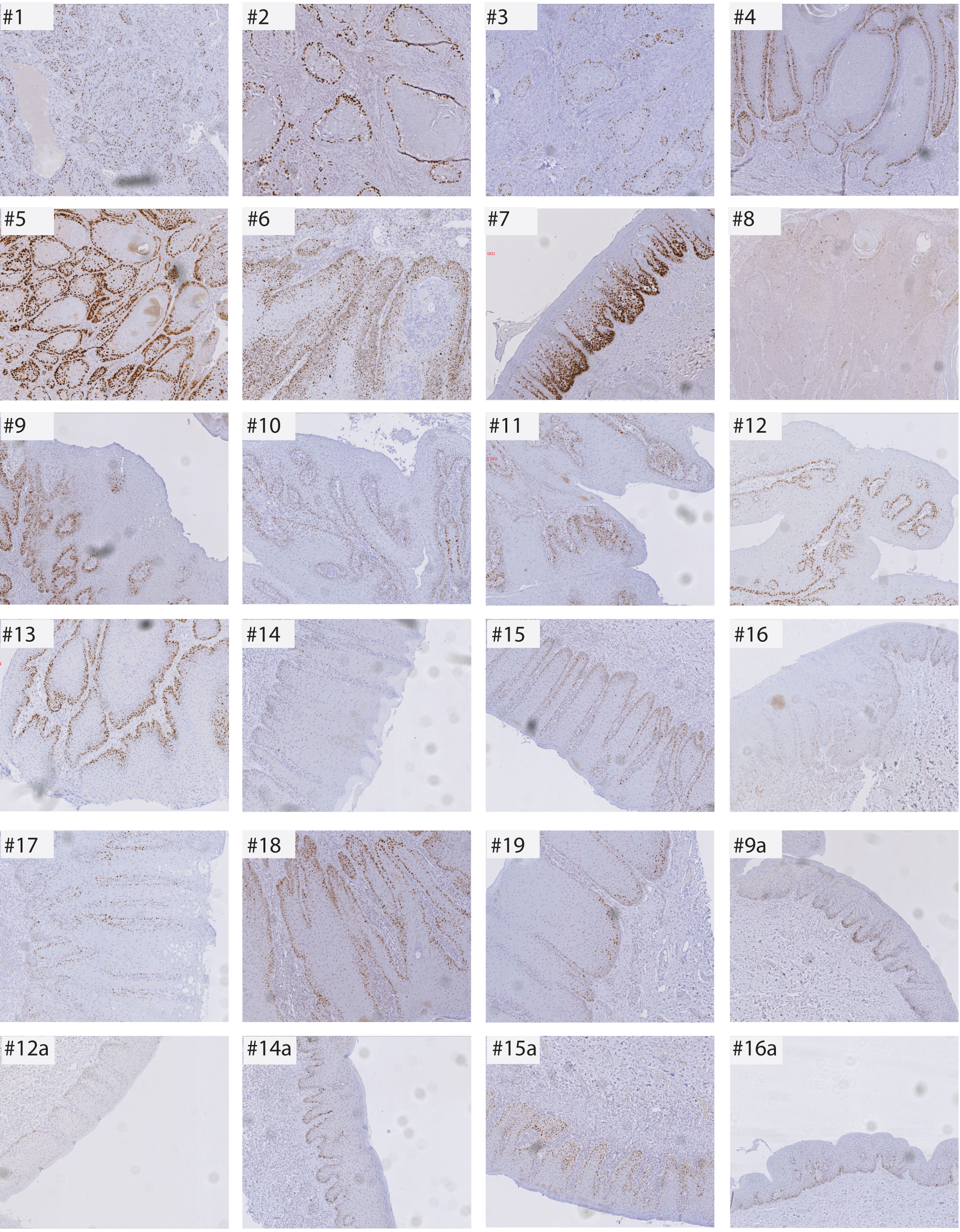

Supplement: Supplementary file 3 — Additional file 3: Figure S3. p53 immunostaining in all samples. One representative p53 immunostaining image photographed using 4x objectives of each sample used in this study are shown (#1–3 SCC, #4, #5 early SCC, #6–8 CIS, #9–13 Papilloma, #14–19 Hyperplasia, #9a, #12a, #14a, #15a and #16a normal adjacent skin). [file 12917_2019_2097_MOESM3_ESM.jpg]

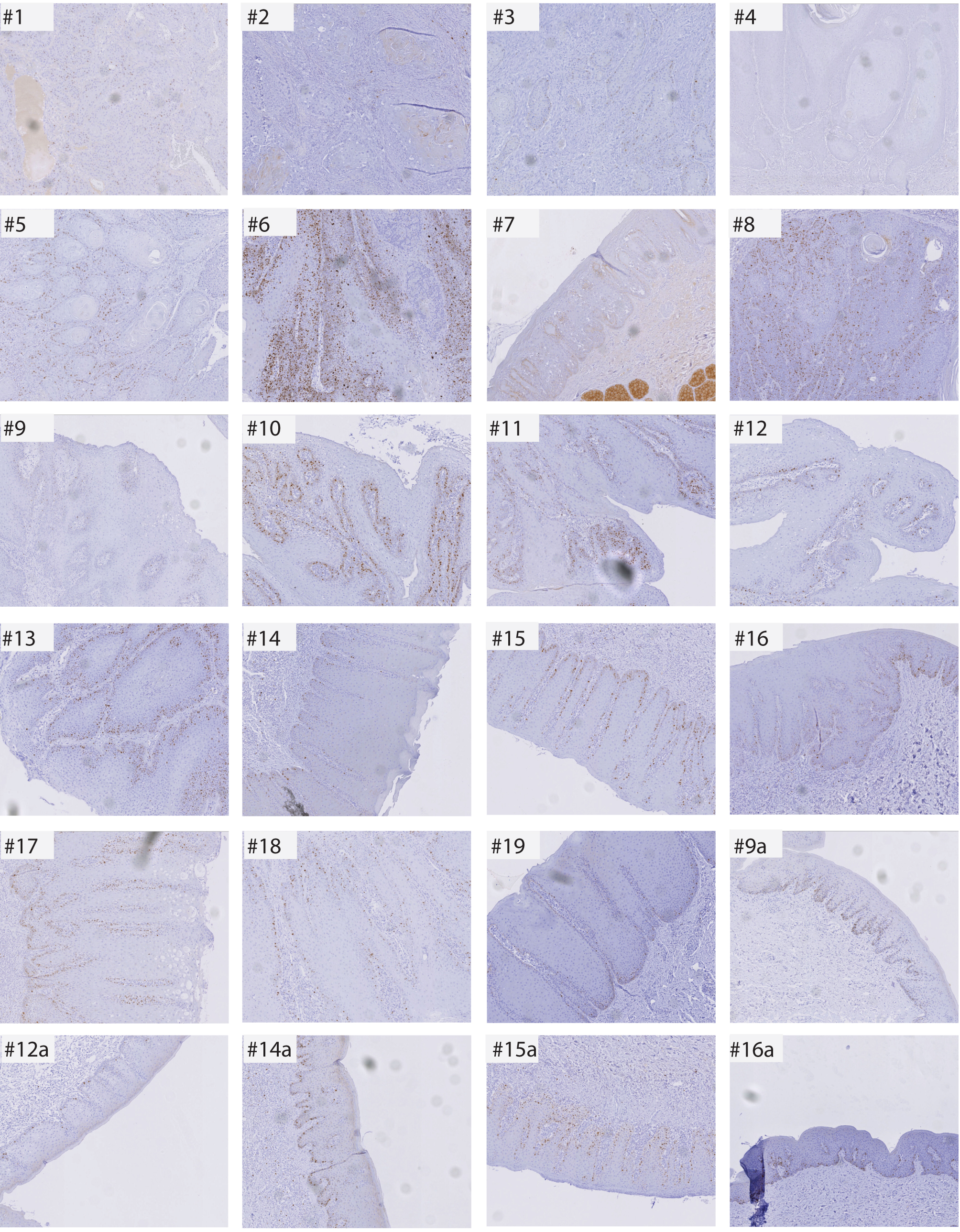

Supplement: Supplementary file 4 — Additional file 4: Figure S4. Ki67 immunostaining in all samples. One representative Ki67 immunostaining image photographed using 4x objectives of each sample used in this study are shown (#1–3 SCC, #4, #5 early SCC, #6–8 CIS, #9–13 Papilloma, #14–19 Hyperplasia, #9a, #12a, #14a, #15a and #16a normal adjacent skin). [file 12917_2019_2097_MOESM4_ESM.jpg]

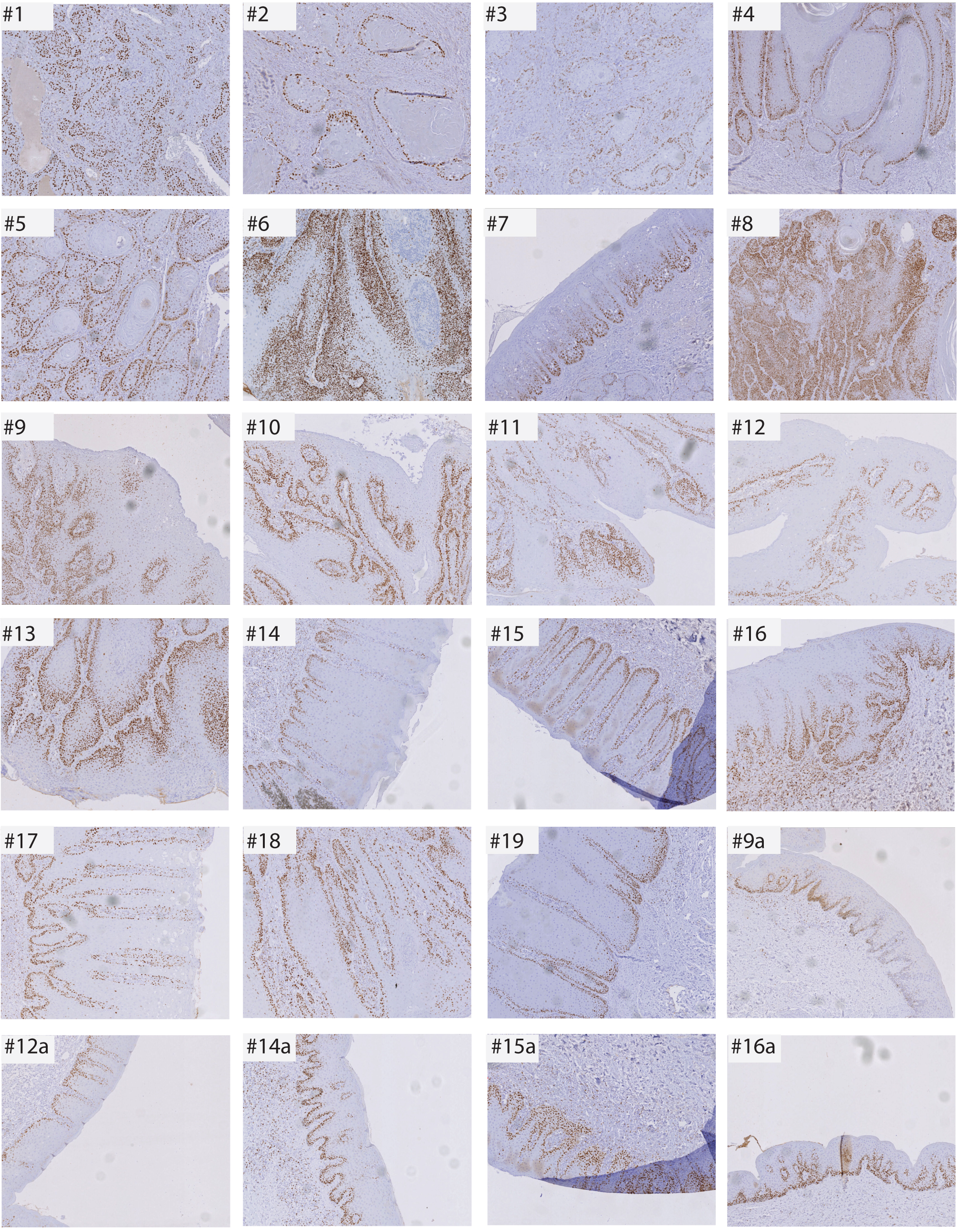

Supplement: Supplementary file 5 — Additional file 5: Figure S5. MCM7 immunostaining in all samples. One representative MCM7 immunostaining image photographed using 4x objectives of each sample used in this study are shown (#1–3 SCC, #4, #5 early SCC, #6–8 CIS, #9–13 Papilloma, #14–19 Hyperplasia, #9a, #12a, #14a, #15a and #16a normal adjacent skin). [file 12917_2019_2097_MOESM5_ESM.jpg]
